# Progress in Diseases of the Blood

**Published:** 1902-12-06

**Authors:** 


### Progress in Diseases of the Blood.

**Blood Counting.**—A. E. Wright[1] describes many ingenious methods for measuring and counting specimens of blood and bacteria. Thus, instead of taking a "platinum wire loopful" of a culture he employs the quantity which is taken up by a capillary tube marked as holding a definite amount. He easily graduates these tubes by a standard pipette from Gower's hæmocytometer which he fills with mercury to the point desired. He next pours this mercury into a series of drawn-out glass tubes and marks on the outsides the points to which the mercury extends, and thus has a number of exact measuring tubes of the cheapest kind. Again, he shows how to dispense with the standardised pipettes and ruled stage for blood counts by making definite dilutions and taking a fixed field as the unit for measurement. Another useful note shows how to make numbers of measured coagulation tubes for testing the length of time which the blood takes to clot. These tubes meet a great want since hæmorrhages tend to occur when the blood is deficient in calcium salts, and there is no easy method of ascertaining this condition, nor of finding out when enough calcium chloride has been given to prevent it. Moreover, an excess of the salt actually lowers the power of clotting. Hence, without testing, we may actually do harm in giving the calcium chloride, nor can we know when there is a dangerous tendency to thrombosis which should be remedied by giving citric acid or soap. Thus the danger of gelatin injections for aneurysm may be obviated by thus testing the blood. The hæmorrhages in typhoid or phthisis may often be controlled, or the thrombosis in typhoid or puerperal or aged patients be prevented, and the occurrence of chilblains and of urticaria after eating much acid fruit be relieved if the clotting power of the blood is known. This can be done most easily if some fine-drawn tubes are taken and the part marked where 5 c.m. of mercury (measured by a Gower's pipette) occupies just 5 m.m. The mercury in each tube is then allowed to fall a centimetre or so lower, its upper and lower limits again marked, and the tube broken at the lowest marking. When these tubes are used, blood is carefully drawn up till it reaches in each the middle marking, showing that 5 c.m. are present, and it is then shaken into the calibrated portion. The tubes are then left for three minutes and upwards and the blood blown out from them one by one over white filter paper to detect the first shred of fibrin. If the air of the room is not about 65° to 66° Fahr., the tubes should be placed in a glass of water of that temperature. W. G. Savage[2] recommends a modification of Stengel's plan of counting the leucocytes. He uses the small pipette of Thoma-Zeiss, as in counting red cells, fills it up to the mark 1, dilutes with a weak staining fluid, and enumerates the red ones. He next draws out the eye-piece till a diameter of the field is just spanned by an exact number of squares. If this number is represented by $x$, and the average number of white cells in such a field be $y$, then the white cells in a cubic millimetre of blood is given by the formula $\frac{5{,}600{,}000\,y}{11\,x^2}$. Thus with any microscope or eye-piece it is only necessary to count the white cells in a number of fields of vision whose diameters are each equal to one fixed number of squares. The count is made too over the same ground as that of the red cells, which ensures accuracy, and we are saved the trouble of using two tubes and of drawing much blood. Finally, the confusion caused under the old plan by the incomplete destruction of red cells by the acetic acid is thus avoided.

**The Biological Test for Blood.**—G. H. F. Nuttall[3] gives the results of his investigations as to the certainty of distinguishing the blood of one animal from another by the precipitation produced by the serum proper to the species. Thus human blood can be differentiated from that of other mammals. "Antihorse" serum, when tested with the blood of 499 other animals, reacted only with the blood of the horse and the ass, "antilobster" serum only gave a precipitum with lobster blood, and a faint clouding with that of the crayfish and five species of crabs out of 500 tests. The certainty of the results depends on a definite strength of the anti-serum and on a fixed time limit being observed. With overstrong anti-serum, or a lengthened time some degree of clouding with other varieties of animals is obtained. From this fact it has been found possible to measure the "blood relationship" of different species. Thus clouding is produced in various degrees in all mammalian blood by the antiserum of a mammal, but none in avian or crustacean ones. Hence there are special properties in blood peculiar to groups and genera, besides that which is peculiar to each species.

**Chloroma.**—This is a rare disease, found chiefly in male children. The red cells may be reduced as low as 1,000,000, and some may be nucleated while the hæmoglobin is much reduced. The whites vary and may reach 300,000, but the polymorpho-nuclear and small lymphocytes are much reduced, and myelocytes may be present with numerous large lymphocytes.[4] There are hæmorrhages under the skin and mucous membranes, and sometimes green nodules, which also grow from the bones of the skull, especially the orbit. This frequently gives rise to protrusion of the eyeball, with blindness and optic neuritis. The spinal cord may be compressed by similar growths, which may also invade the heart, liver, spleen, and kidneys. The lymphatic glands are enlarged and of the same green tint. They contain numbers of large mononuclear cells. It is disputed whether the disease is related to lymphatic leukæmia or to lymphosarcoma, since the excess of white cells is not always present and may be found only a short time before death. Only some 25 cases have been observed, but G. H. Dunlop and Byrom Bramwell have lately brought forward instances in a boy of 5 years and a man of 25. Dunlop[5] thinks that the green colour of the tumours is due to a substance, allied to lipochrome, which becomes oxydised on exposure to light, for the colour fades on exposure to air, or when immersed in fluids which have no action on fats. Dock believes the colour is due to some form of fat, while Bramwell is inclined to refer it to a blood pigment, but thinks that it may be due to various causes in different cases, and that it is absent altogether in undoubted instances of the disease. The tumours seem to start from the periosteum of the head and face, and though secondary deposits are numerous, the brain and nervous system are never invaded by them. He[6] points out that the blood state is very like that in acute lymphatic leukæmia. There is the same huge excess of lymphocytes, and especially of the large lymphocytes; the total number of leucocytes may or may not be increased, and the marrow, spleen, and lymph glands are infiltrated with lymphatic deposits. Moreover, in a case of acute L. leukæmia Bramwell found that some of the glands were green, and that the gums, tonsils, and larynx were infiltrated just as in his case of chloroma.

[1] Lancet, July 5. [2] Ibid., Sept. 27. [3] Brit. Med. Jour., April 5. [4] Birm. Med. Rev., June. [5] Brit. Med. Jour., May 3. [6] Ibid, Feb. 22.

*(To be continued.)*

